# Supplementary material for: The Cytotoxic Effects of Human Mesenchymal Stem Cells Induced by Uranium
Source: Biology (Basel). 2024 Jul 16;13(7):525. doi: 10.3390/biology13070525 (PMC11274140; doi:10.3390/biology13070525)

Supplementary

Figure S1. Cell viability impacted by a gradient concentration lindane. Cells were cultured with a gradient concentration lindane respectively for 2 h, then cells were harvested and adjusted to the same cell concentration with incubation medium. 100 µl cell suspension solution was added in 96-well culture plate with three duplicate. CCK-8 assay was performed at 24 h post reseeding. Data were normalized to control group.

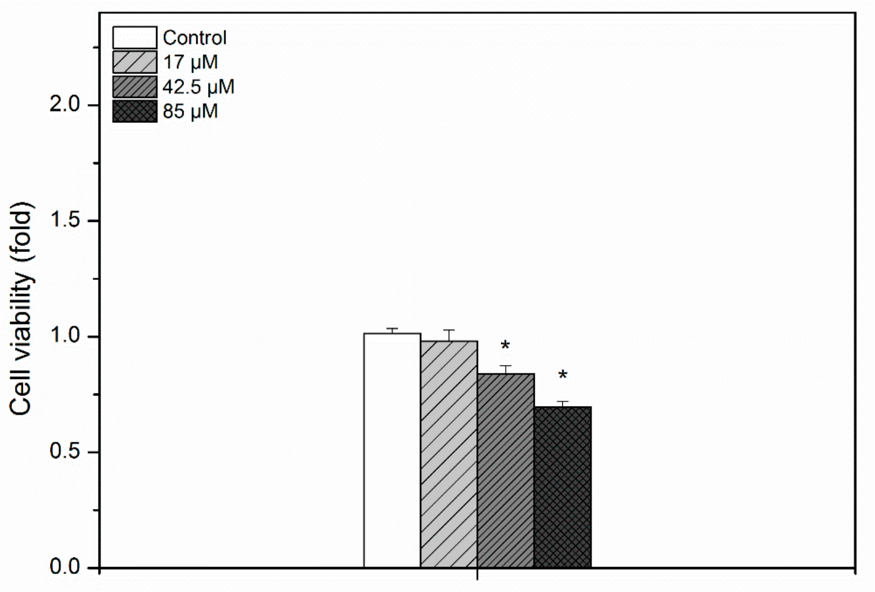

Figure S2. The representative picture of cells with γH2AX foci produced by uranyl nitrate with different concentration. After exposed to uranyl nitrate for 24 h, cells were sampled at 30 min or 24 h post treatment. It was stained with γH2AX primary antibody and then labeled with cy3-conjugated secondary antibody. The red fluorescence foci showed DNA double strand breaks and blue fluorescence showed cell nucleus. Bar = 10 µm.

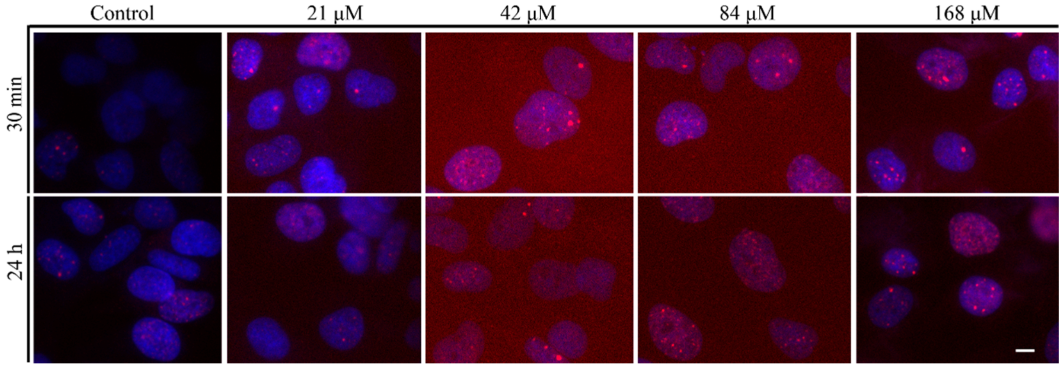

Figure S3. The original imaging of protein bands for connexin32. After exposed to different concentration uranyl nitrate for 24 h, cells were sampled at 30 min or 24 h post treatment.

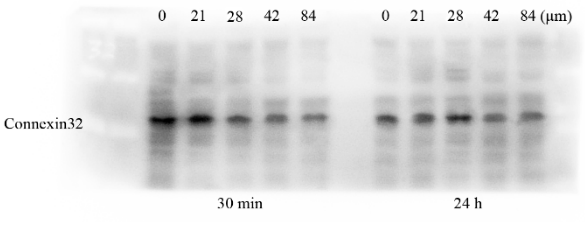

Figure S4. The original imaging of protein bands for connexin43. After exposed to different concentration uranyl nitrate for 24 h, cells were sampled at 30 min or 24 h post treatment.

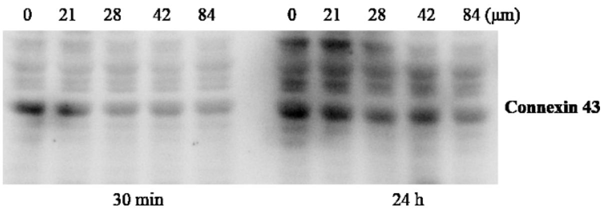

Supplement: Supplementary file 1 [file biology-13-00525-s001.zip › biology-3069582-supplementary.pdf]
